# Ultrasonographic features of a pulmonary artery pseudoaneurysm secondary to a migrating intrathoracic grass awn in a dog: a case report

**DOI:** 10.1186/s12917-026-05517-5

**Published:** 2026-04-28

**Authors:** Michał Gruss, Anna Łukomska, Maciej Głębicki, Maciej Gogulski, Roland Kozdrowski

**Affiliations:** 1 Department of Internal Diseases and Diagnostics, Poznan University of Life Sciences, Szydłowska 50, Poznań, 60-637 Poland; 2 Department of Preclinical Sciences and Infectious Diseases, Poznan University of Life Sciences, Wołynska 35, Poznań, 60-637 Poland; 3 University Centre for Veterinary Medicine, Szydłowska 43, Poznań, 60-656 Poland

**Keywords:** Lung, Ultrasonography, Vascular, Pulmonary artery, Pseudoaneurysm, Foreign body

## Abstract

**Background:**

Pulmonary artery pseudoaneurysm (PAP) is a rare but well-documented vascular abnormality in humans and can be life-threatening. In companion animals, data on PAP are scarce. While the clinical course and imaging features of migrating intrathoracic grass awns in dogs are well documented, they have not previously been associated with PAP. Lung ultrasonography (LUS) is increasingly used in veterinary medicine, with multiple sonographic patterns reported for pulmonary disease, including disorders caused by migrating grass awns. This report describes an unprecedented ultrasonographic appearance of a peripheral PAP secondary to a migrating intrathoracic grass awn in a dog.

**Case presentation:**

A 10-year-old spayed female Yorkshire Terrier was evaluated for a 3-day history of lethargy, inappetence, and coughing. Thoracic radiography revealed an interstitial-to-alveolar pattern in the right caudal lung lobe, and echocardiography identified myxomatous mitral valve disease. LUS demonstrated a pulsatile, hypoechoic lesion in the caudodorsal region of the right caudal lung lobe, in continuity with a pulmonary artery branch. The lesion had an oval to pear-shaped appearance, and color Doppler showed a “yin–yang” pattern of swirling intraluminal flow. A subtle hyperechoic interface casting an acoustic shadow, suggestive of a foreign body, was visible adjacent to the deep wall of the lesion. Despite empirical treatment for pneumonia, the dog deteriorated and was euthanized. Postmortem LUS of the excised lung supported the presence of a vegetal foreign body within the lesion. Gross and histopathological examination established a diagnosis of peripheral PAP associated with necrotizing, suppurative pneumonia and an intrabronchial grass awn.

**Conclusions:**

This case suggests that PAP may represent a potential sequela of intrabronchial grass awn migration in dogs. On LUS, a hypoechoic, pulsatile intrapulmonary lesion with a “yin–yang” Doppler flow pattern should prompt consideration of PAP. Although further studies are needed, careful assessment of similar ultrasonographic findings may facilitate the recognition of clinically relevant vascular lesions in dogs.

**Supplementary Information:**

The online version contains supplementary material available at 10.1186/s12917-026-05517-5.

## Background

Pulmonary artery pseudoaneurysm (PAP) is a rare but potentially fatal condition in humans, characterized by focal dilatation of the pulmonary artery or its branches [1, 2]. Unlike true aneurysm, which involves dilatation of all three layers of the vessel wall, pseudoaneurysm results from the disruption of the *tunica intima* and possibly the *tunica media*, allowing blood to accumulate within the *tunica adventitia* or surrounding soft tissues [3, 4]. In humans, most PAPs are acquired, typically due to infection, trauma, or malignancy [2]. Reports of PAP in companion animals are scarce; only one publication briefly mentions a pseudoaneurysm of the main pulmonary artery in the necropsy findings of a cat with systemic hypertension and vasa vasorum arteriopathy [5]. 

Grass awns are a well-recognized cause of respiratory disease in dogs. Their migration into the lungs or pleural space can result in severe complications, including lobar pneumonia, abscess formation, pneumothorax, and pyothorax [6, 7]. While the clinical and pathological features of diseases caused by migrating grass awns are well documented, vascular complications associated with their migration have been reported only rarely, and not within the thoracic cavity. Among these, the previously reported grass awn–associated pseudoaneurysms in dogs involved the celiac artery and the common carotid artery [8, 9]. 

Lung ultrasonography (LUS) is increasingly used in small animal practice to assess pulmonary disease, including disorders caused by migrating grass awns [7, 10]. Reports describing the sonographic features of PAP in either humans or companion animals appear to be lacking. Herein, we describe the sonographic appearance of a peripheral PAP secondary to a migrating intrathoracic grass awn in a dog.

## Case presentation

### Clinical features

A 10-year-old spayed female Yorkshire Terrier was evaluated for a 3-day history of lethargy, inappetence, and coughing. The dog had no prior medical history and had received routine preventive care. On physical examination, the dog weighed 4.1 kg, appeared adequately hydrated, and had a rectal temperature of 39.6 °C. The mucous membranes were pink and moist with a normal capillary refill time. Heart rate was 110 bpm, and respiratory rate could not be accurately assessed due to panting. Pulmonary auscultation revealed harsh lung sounds in the right caudodorsal lung field. Cardiac auscultation identified a grade III/VI systolic murmur. Femoral pulses were strong and synchronous. Mild abdominal discomfort was elicited on palpation. Peripheral lymph node abnormalities were not detected.

A complete blood count revealed moderate leukocytosis (23.41 × 10⁹/L; reference interval, 6–16 × 10⁹/L) with neutrophilia and left shift. In-house serum biochemistry, including liver and renal parameters, protein and lipid profiles, and electrolyte concentrations, revealed no abnormalities. Quantitative canine pancreatic lipase concentration was within the reference range. Thoracic radiographs showed an interstitial to alveolar pattern in the right caudal lung lobe and left atrial enlargement. Other thoracic structures were within normal limits. Echocardiography demonstrated myxomatous mitral valve disease with secondary volume overload of the left-sided cardiac chambers, consistent with ACVIM stage B2.

### Ultrasonographic findings

**Fig. 1:**
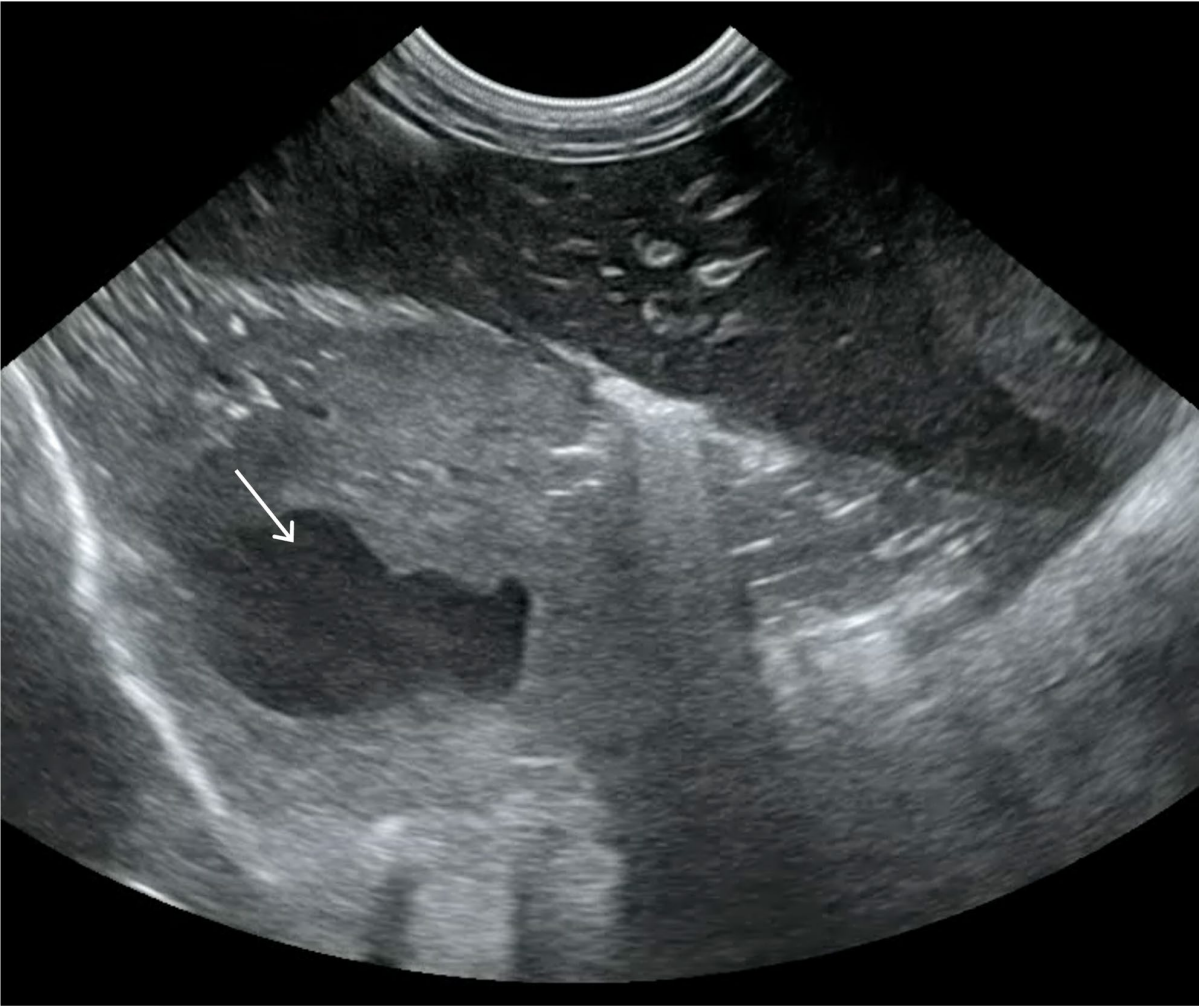
Transdiaphragmatic ultrasonographic image of the consolidated lung, with a hypoechoic lesion (arrow) consistent with the lumen of a pulmonary artery pseudoaneurysm (PAP). The liver and the cranial pole of the right kidney are seen in the near field. Left on the image is cranial

**Fig. 2:**
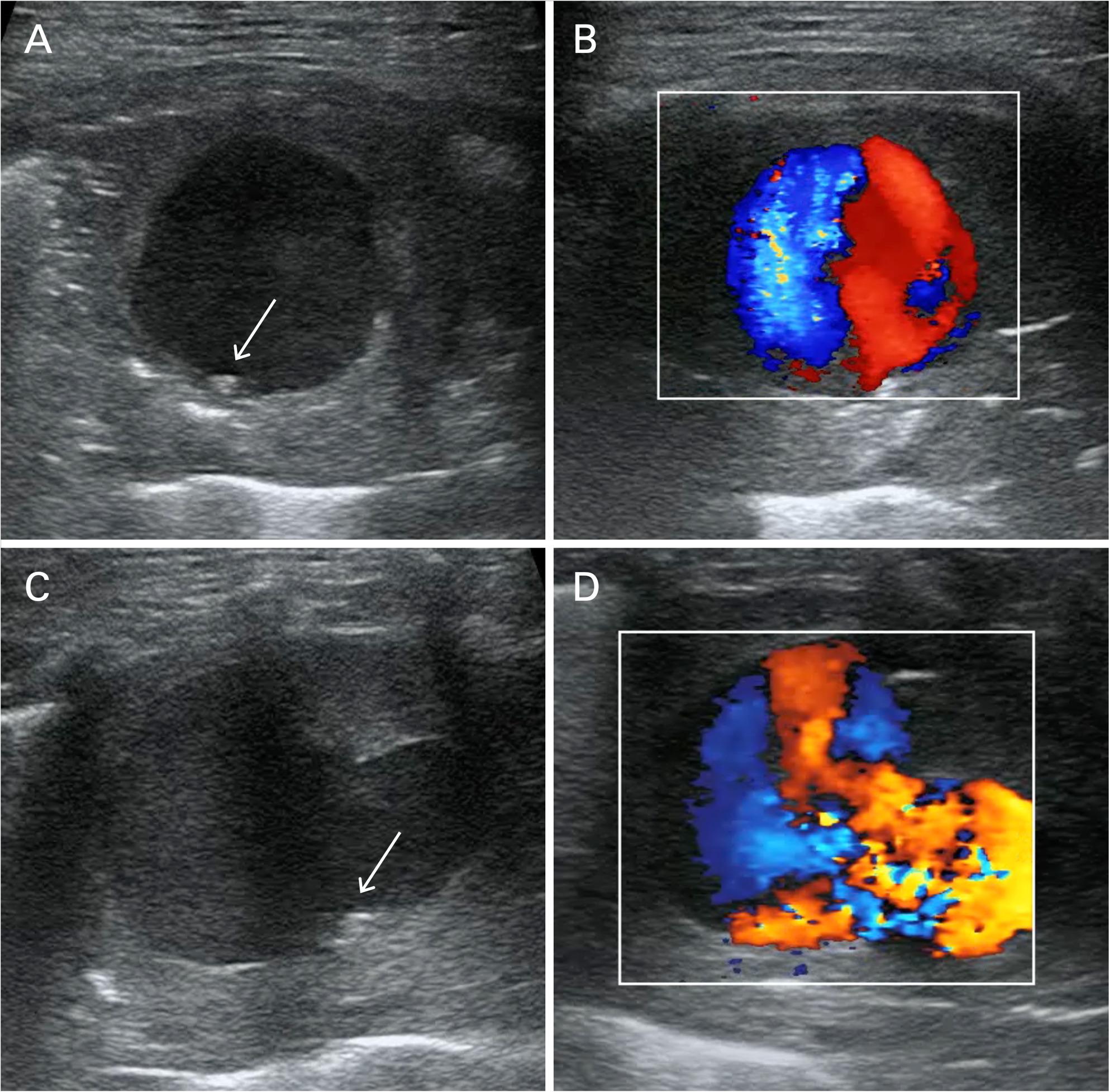
Ultrasonographic appearances of PAP obtained at different insonation angles. In B-mode, the lesion is hypoechoic and its shape ranges from oval (**A**) to irregular, pear-shaped (**C**). In both B-mode panels, the arrow indicates the hyperechoic interface of the grass awn. The corresponding color Doppler images show swirling intraluminal flow with a “yin–yang” pattern (**B**, **D**). Left on the image is dorsal in A and B, and cranial in C and D

LUS revealed a pulsatile, hypoechoic lesion in the caudodorsal region of the right lung (Fig. 1; Additional file 1). The lesion had a maximum length of approximately 3 cm, and its shape varied with the insonation angle, ranging from oval to irregular, pear-shaped form (Fig. 2 A, C). Although the wall of the lesion appeared thickened, it was difficult to distinguish it clearly from the surrounding consolidated lung tissue. The lumen appeared continuous with an arterial vessel visualized in the far field. Color Doppler investigation demonstrated a characteristic “yin–yang” pattern of mixed flow signals within the lesion, consistent with swirling intraluminal blood flow (Fig. 2 B, D; Additional file 2). A subtle hyperechoic structure was observed within the lesion, adjacent to its deep wall, casting a discrete acoustic shadow (Fig. 2A, C). The lung surrounding the pulsatile lesion appeared consolidated. Craniomedially, dynamic air bronchograms were observed within the consolidated parenchyma (Fig. 2A). At the level of the consolidation, the pleura was irregularly thickened, and the sliding sign was absent. In the caudodorsal portion of the lung, the parenchyma appeared uniformly hypoechoic, lacked visible bronchograms, but preserved a normal vascular pattern—features consistent with atelectasis. The described sonographic changes were confined to the right caudal lung lobe. The remaining areas of the right lung and the entire left lung appeared normal. Abdominal ultrasonography performed with the dog standing to minimize respiratory effort revealed mild gallbladder wall thickening (1.7 mm; upper reference limit, 1.3 mm [11]) with a small amount of mobile sludge, as well as mild renal contour irregularities with partial obliteration of the corticomedullary junction. These findings were considered clinically irrelevant.

Based on the clinical and imaging findings, pneumonia with a focal pulmonary arterial anomaly and possible foreign body involvement was suspected. The owner declined further diagnostic and interventional procedures, and empirical treatment for pneumonia was initiated using intravenous antibiotics along with supportive care. Therapy for mitral valve disease was concurrently initiated. Despite this treatment, the dog’s condition deteriorated over the next two days, with marked progression of respiratory distress. In view of the poor prognosis, euthanasia was elected at the owner’s request. Without interrupting oxygen therapy, the dog was premedicated intravenously with methadone hydrochloride (0.4 mg/kg) and dexmedetomidine hydrochloride (0.8 mg/kg). Once adequate sedation was achieved, sodium pentobarbital was administered intravenously at an overdose of 100 mg/kg to complete the procedure in accordance with accepted professional guidelines [12]. 

### Postmortem ultrasonography and histologic examination

**Fig. 3:**
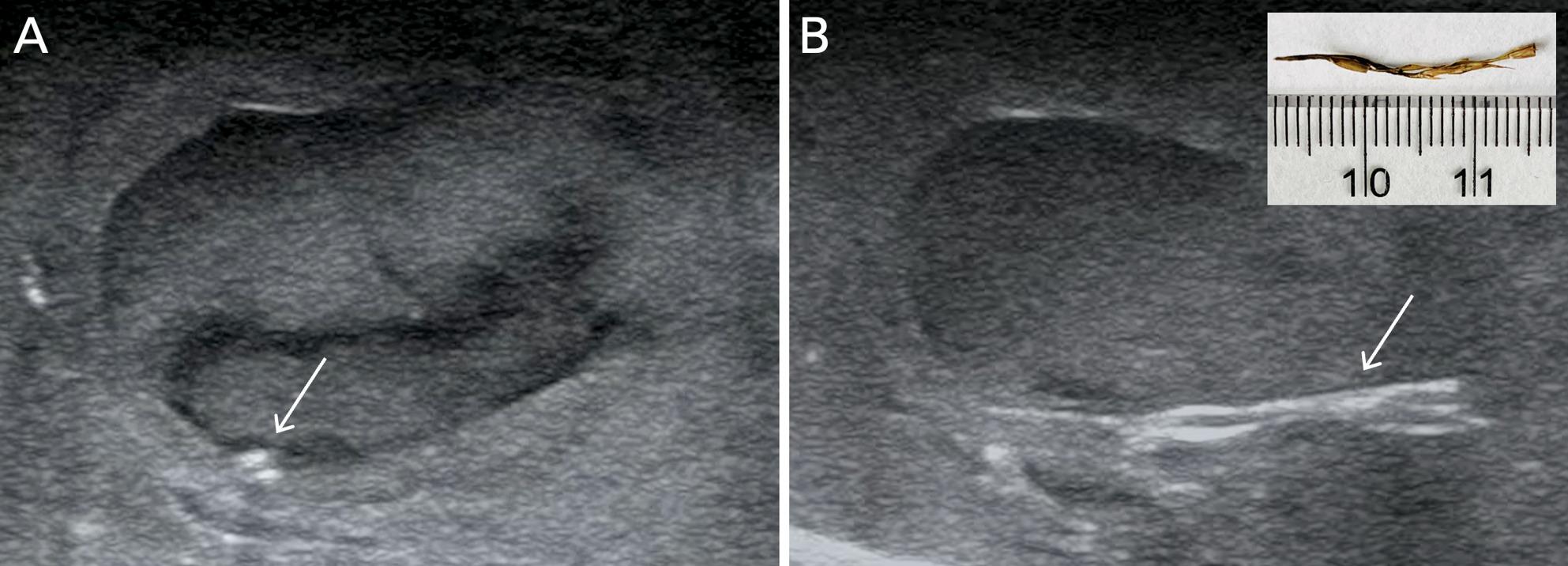
Postmortem ultrasonographic images of the excised lung, with the probe placed directly on the visceral pleura at the site of the PAP. The wall of the PAP is more distinct, and the lumen shows heterogeneous echogenicity consistent with clot formation. Arrows indicate the hyperechoic interface of the foreign body in the transverse (**A**) and longitudinal (**B**) planes. This ultrasonographic finding corresponds to the macroscopic appearance of the grass awn retrieved during necropsy from a small bronchus adjacent to the PAP (B, inset)

Ultrasonographic examination of the excised lung was performed prior to dissection. By adjusting the insonation angle, the hyperechoic linear structure that had been faintly visible during the previous examination could be visualized in multiple planes, confirming the suspicion of a vegetal foreign body located in the deeper portion of the lesion (Fig. 3).

**Fig. 4:**
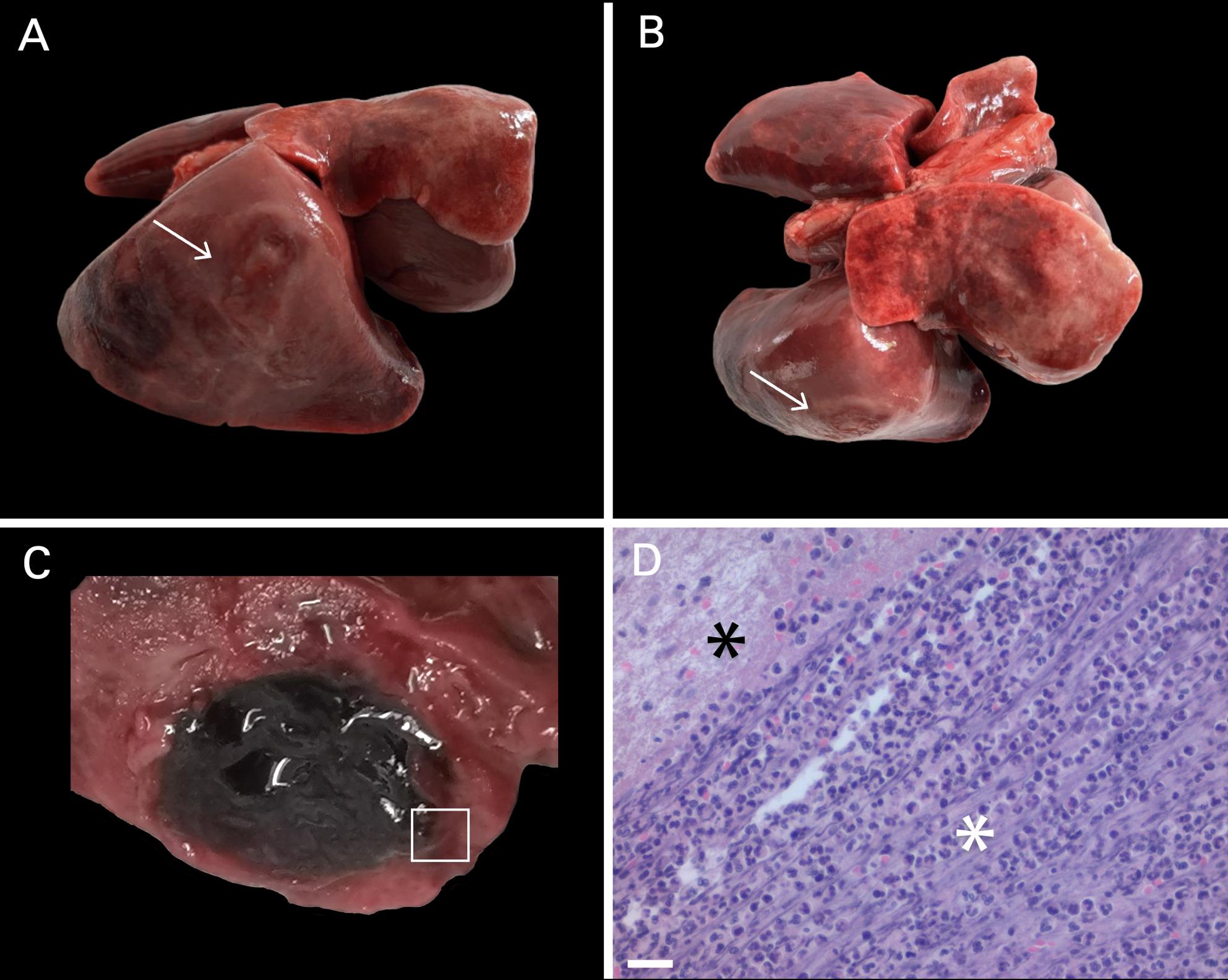
Gross and microscopic structure of the lesion. Gross images show the costal (parietal) surface of the right lung from the right-lateral perspective (**A**) and a dorsal (top-down) view (**B**); the irregularity and bulging of the visceral pleura at the site of the PAP in the caudal lobe of the right lung are evident (arrow). A transverse section through the PAP (**C**) shows a distinct intraparenchymal clot; the histologic field in D corresponds to the white rectangle in C. Photomicrograph (**D**) demonstrates suppurative and necrotizing exudate (white asterisk) adjacent to early hematoma (black asterisk). H&E stain; scale bar = 20 µm

On gross examination, an intrapulmonary artery emptied into a blood-filled cavity containing clotted material and surrounded by a thick fibrous wall. This cavity caused a bulging of the pleural surface of the right caudal lung lobe (Fig. 4A–C). Upon dissection, a grass awn (Fig. 3B, inset) was retrieved from a small bronchus adjacent to the lesion. On histology, a large central hemorrhagic area was surrounded by thick fibrinous and inflammatory deposits, suggestive of early hematoma formation (Fig. 4D). Mild thickening of the tunica media in small- and medium-sized arterioles was noted. The pulmonary parenchyma was largely replaced by a focally extensive necrotizing and inflammatory process, in which the alveolar septa and alveolar spaces were infiltrated by neutrophils, fibrin, and cellular debris. The final diagnosis was peripheral pulmonary artery pseudoaneurysm associated with pyogenic pneumonia and an intrabronchial grass awn.

## Discussion and conclusions

Sonographic features of pseudoaneurysms in anatomical locations other than the lungs are well established in human medicine [13] and have also been reported in a limited number of veterinary cases, including pseudoaneurysms of the celiac, mesenteric, and femoral arteries [4, 8, 14]. In B-mode ultrasonography, pseudoaneurysms typically appear as anechoic to mixed heterogeneous collections located adjacent to a supplying artery [13, 15]. This description aligns with our findings: the PAP appeared as a hypoechoic pulsatile structure in direct continuity with a pulmonary artery. This sonographic appearance corresponded well with the histologic findings, in which the central hypoechoic region represented extravasated blood and the wall consisted of fibrous tissue (Fig. 4). While pulsation is not considered a primary sonographic criterion for pseudoaneurysm diagnosis, it often becomes apparent due to the real-time nature of ultrasonography [15]. 

Nonetheless, grayscale imaging alone does not provide definitive evidence of pseudoaneurysm, as similar findings may occur in hematomas or other cystic lesions [13]. Therefore, Doppler investigation is usually essential for diagnosis. On color Doppler, the swirling flow pattern generated by systolic inflow and diastolic outflow produces the classic “yin–yang” sign [13, 16], which was clearly observed in our patient. However, this sign may also be observed in saccular aneurysms, which should be considered in the differential diagnosis [13]. Spectral Doppler can help differentiate between these two entities. When applied at the neck of a pseudoaneurysm, it typically reveals a bidirectional turbulent flow pattern—the characteristic “to-and-fro” waveform. The “to” component corresponds to arterial inflow during systole, while the “fro” reflects retrograde outflow during diastole [13]. At the time of the examination, we were not fully aware of the diagnostic significance of this feature. Although spectral Doppler was applied and a waveform resembling a ‘to-and-fro’ pattern was observed, the sample gate was not positioned directly at the neck of the pseudoaneurysm. Consequently, this finding was not included in the formal diagnostic interpretation.

In this patient, a hyperechoic interface casting acoustic shadow was observed adjacent to the pseudoaneurysm—features suggestive of foreign material. Grass awn foreign bodies typically exhibit a characteristic ultrasonographic appearance, described as a hyperechoic interface with a spindle-shaped or tapering, cigar-shaped outline [17]. Ultrasonography is useful for diagnosing grass awn–associated conditions in accessible anatomical regions, such as interdigital spaces [18]. However, in less accessible locations or when the foreign body is small, direct ultrasonographic visualization may not be feasible [19]. In one study, 29 dogs were diagnosed with migrating pleural grass awns and 10 were located within the lung parenchyma [7]. LUS enabled the detection of only 13 foreign bodies in the pleural space and 7 within the lung parenchyma. Similar findings were reported in a previous study, where ultrasonography allowed identification of 4 out of 8 intrathoracic grass awns [10]. In this case, a foreign body was suspected based on sonographic findings, but it appeared equivocal due to its subtle nature and its proximity to consolidated lung parenchyma, where similar hyperechoic foci might represent air bronchograms. Notably, visualization of the foreign material improved when the probe was placed directly on the pleural surface of the excised lung. This likely resulted from improved image quality obtained by scanning a motionless lung and optimizing the insonation angle, which allowed visualization of the foreign body in the longitudinal plane.

In cases of intrapulmonary grass awn migration in dogs, authors have reported sonographic signs such as pulmonary consolidation, focal pleural adhesions (manifested as absence of the glide sign), and focal pleural effusion. All of these features—except pleural effusion—were also present in this case. Although pleural adhesions were not definitively confirmed on necropsy, the visceral pleura overlying the pseudoaneurysm appeared irregular and disrupted. Additionally, the lung surface in this area was protruding, which may explain the absence of the glide sign observed during ultrasonographic examination. The most caudal area of the affected lung lobe appeared uniformly consolidated while maintaining a normal vascular pattern. Given the central location of the pseudoaneurysm, resorptive atelectasis likely developed secondary to the lesion. These areas may also reflect parenchymal necrosis and inflammatory infiltrates, both prominent on histologic examination.

In human medicine, computed tomography angiography and catheter-directed angiography are regarded as the primary imaging techniques for diagnosing PAP [1, 20]. Chest radiographs may show focal lung consolidation or a solitary pulmonary nodule [1, 20]. In this case, a focal interstitial-to-alveolar pattern was observed, which is also a common radiographic finding in dogs with intrathoracic migrating grass awns, as reported previously [10]. The most likely reason a distinct nodule or mass was not identified in this case was the superimposition of the pseudoaneurysm and surrounding inflamed lung tissue, both of which take on similar radiographic soft tissue opacity. The lack of information on use of ultrasonography in diagnosis of PAP in human medicine likely reflects both the widespread availability of advanced imaging techniques and the inherent limitations of LUS in evaluating intrathoracic vascular abnormalities. It is essential to acknowledge the limitations of LUS in assessing intrapulmonary lesions, particularly its dependence on adequate acoustic windows.

The location of the PAP in the right caudal lung lobe likely reflects the foreign body’s final position and is consistent with published reports. In a study evaluating the distribution of bronchial grass awns in dogs using bronchoscopy, 76.6% were found in the right lung, with the right caudal lobe accounting for 25.5% of all cases [21]. In human medicine, PAPs are typically classified by location as either proximal or peripheral [2]. The latter involve intrapulmonary branches of the pulmonary arteries, and based on this classification, the lesion observed in our patient falls into this category. This distinction is clinically relevant, as the thinner, more fragile structure of peripheral vessels makes them particularly prone to rupture, rendering such lesions potentially life-threatening.

Acquired PAPs are most commonly associated with trauma, infection, vasculitis, or neoplasia [2]. In this case, disruption of the arterial wall may have resulted either from direct mechanical injury caused by the intrabronchial grass awn, from adjacent necrotizing pneumonia, or from a combination of both. Although direct vascular injury could not be confirmed histologically—since the site of disruption was not identified and the foreign body had been removed during gross examination—ultrasonography demonstrated the foreign body within the pseudoaneurysm, supporting a potential causal relationship. Nevertheless, it remains possible that local inflammation alone, without direct mechanical penetration, may have been sufficient to initiate pseudoaneurysm formation.

Several limitations should be acknowledged. Most notably, the diagnosis was based solely on ultrasonographic findings corroborated by postmortem confirmation; no advanced imaging modalities were performed, which precluded direct modality comparison with the extensive human medical literature. The precise clinical significance of the PAP in this case remains uncertain. It is unclear whether the dog’s deterioration resulted from foreign body–associated pneumonia, progression of the pseudoaneurysm, or an interplay between both processes. Furthermore, this is a single case report, and caution should be exercised when extrapolating these findings to broader clinical scenarios. Additional studies are necessary to elucidate the pathophysiological relevance of PAP and evaluate the diagnostic reliability of LUS for detecting this rare lesion.

## Supplementary Information

Supplementary Material 1: Additional file 1.File format: MP4Title of data: B-mode ultrasonographic cine loop of a pulmonary artery pseudoaneurysm. Description of data: B-mode ultrasonographic features of a pulmonary artery pseudoaneurysm secondary to a migrating grass awn in a dog, with audio narration describing the key ultrasonographic features.

Supplementary Material 2: Additional file 2.File format: MP4 Title of data: Color Doppler ultrasonographic cine loop of a pulmonary artery pseudoaneurysm. Description of data: Color Doppler ultrasonographic features of a pulmonary artery pseudoaneurysm secondary to a migrating grass awn in a dog

## Data Availability

Clinical data and images from this case report are available from the corresponding author upon reasonable request.

## Abbreviations

PAP: Pulmonary Artery Pseudoaneurysm
LUS: Lung Ultrasonography
